# Serotonin Improves High Fat Diet Induced Obesity in Mice

**DOI:** 10.1371/journal.pone.0147143

**Published:** 2016-01-14

**Authors:** Hitoshi Watanabe, Tatsuya Nakano, Ryo Saito, Daisuke Akasaka, Kazuki Saito, Hideki Ogasawara, Takeshi Minashima, Kohtaro Miyazawa, Takashi Kanaya, Ikuro Takakura, Nao Inoue, Ikuo Ikeda, Xiangning Chen, Masato Miyake, Haruki Kitazawa, Hitoshi Shirakawa, Kan Sato, Kohji Tahara, Yuya Nagasawa, Michael T. Rose, Shyuichi Ohwada, Kouichi Watanabe, Hisashi Aso

**Affiliations:** 1 Cellar Biology Laboratory, Graduate School of Agricultural Science, Tohoku University, 1–1 Tsutsumidori Amamiyamachi, Aoba-ku, Sendai, 981–8555, Japan; 2 Laboratory of Food and Biomolecular Science, Graduate School of Agricultural Science, Tohoku University, 1–1 Tsutsumidori Amamiyamachi, Aoba-ku, Sendai, 981–8555, Japan; 3 Laboratory of Animal Products Chemistry, Graduate School of Agricultural Science, Tohoku University, 1–1 Tsutsumidori Amamiyamachi, Aoba-ku, Sendai, 981–8555, Japan; 4 Laboratory of Nutrition, Graduate School of Agricultural Science, Tohoku University, 1–1 Tsutsumidori Amamiyamachi, Aoba-ku, Sendai, 981–8555, Japan; 5 Animal Science, Department of Biological Production, Tokyo University of Agriculture and Technology, Fuchu-shi, Tokyo, 183–8509, Japan; 6 Dept. of Advanced Medicine and Development, BML Inc., Saitama, 350–1101, Japan; 7 Institute of Biological, Environmental and Rural Sciences, Aberystwyth University, Cardiganshire, SY23 3DA, United Kingdom; Hosptial Infantil Universitario Niño Jesús, CIBEROBN, SPAIN

## Abstract

There are two independent serotonin (5-HT) systems of organization: one in the central nervous system and the other in the periphery. 5-HT affects feeding behavior and obesity in the central nervous system. On the other hand, peripheral 5-HT also may play an important role in obesity, as it has been reported that 5-HT regulates glucose and lipid metabolism. Here we show that the intraperitoneal injection of 5-HT to mice inhibits weight gain, hyperglycemia and insulin resistance and completely prevented the enlargement of intra-abdominal adipocytes without having any effect on food intake when on a high fat diet, but not on a chow diet. 5-HT increased energy expenditure, O_2_ consumption and CO_2_ production. This novel metabolic effect of peripheral 5-HT is critically related to a shift in the profile of muscle fiber type from fast/glycolytic to slow/oxidative in soleus muscle. Additionally, 5-HT dramatically induced an increase in the mRNA expression of peroxisome proliferator-activated receptor coactivator 1α (PGC-1α)-b and PGC-1α-c in soleus muscle. The elevation of these gene mRNA expressions by 5-HT injection was inhibited by treatment with 5-HT receptor (5HTR) 2A or 7 antagonists. Our results demonstrate that peripheral 5-HT may play an important role in the relief of obesity and other metabolic disorders by accelerating energy consumption in skeletal muscle.

## Introduction

Serotonin (5-HT) is a monoaminergic neurotransmitter with activities that modulate central and peripheral functions. The first step in the synthesis of 5-HT from tryptophan depends on the enzyme tryptophan hydroxylase (TPH), which is also the rate-limiting enzyme in its biosynthesis. TPH is known to possess two isoforms, TPH1 and TPH2 [1]. TPH1 is mostly present in the pineal gland, spleen, thymus and intestinal enterochromaffin cells. TPH2 is expressed entirely in neuronal cells, such as in the raphe nuclei of the brain stem. Peripheral 5-HT in TPH1 knockout mice is not able to be replaced with 5-HT synthesized by TPH2 in the central nervous system [2]. Further, it is thought that 5-HT in the periphery cannot pass the blood-brain barrier [3, 4]. Thus, there are two independent systems of organization for 5HT: one in the central nervous system and the other in the periphery. 5-HT affects feeding behavior and obesity in the central nervous system, and close to 2% of the body’s 5-HT is stored there [5–10]. On the other hand, peripheral 5-HT has not been the subject of such intense study, particularly with respect to body fat and lipid metabolism, even though approximately 98% of the body’s 5-HT exists in the periphery.

Accumulating evidence indicates that peripheral 5-HT plays an important role in glucose and lipid metabolism. Recent studies have shown that the level of blood 5-HT and the number of intestine enterochromaffin cells in obese mice was found to be much higher than that in lean mice [11, 12]. Intraperitoneal (i.p.) injection of 5-HT to mice accelerates the metabolism of lipid by increasing the concentration of circulating bile acids [13], and further, that 5-HT regulates fat metabolism and feeding behavior through independent molecular mechanisms in *Caenorhabditis elegans* [14]. Additionally, TPH1 deficient mice have an impaired insulin secretion and significantly higher blood glucose concentrations than wild type animals in glucose tolerance tests [15]. On the other hand, gut-derived serotonin enhances lipolysis in adipocytes through 5-HT receptor (5HTR) 2B and gluconeogenesis in hepatocytes through 5HTR2B [16]. TPH1 deficient mice are protected from obesity and insulin resistance by elevation of brown adipose tissue activity [17]. These studies suggest that 5-HT may be a key factor with regard to glucose and lipid metabolism, fat accumulation and obesity in not only the central nervous system but also the periphery, as demonstrated through the various phenotypes of available knock out mice.

Skeletal muscle has important roles in energy metabolism and glucose utilization, especially during excise. The existence of slow and fast type myosin heavy chain isoforms is observed in normal mature muscle fibers. Slow type muscle fibers have a high concentration of mitochondria and produce energy by oxidative metabolism. In contrast, fast type muscle fibers use glycolysis as the chief ATP source [18, 19]. Peroxisome proliferator-activated receptor (PPAR) γ coactivator 1 a (PGC-1a), is identified as a nuclear receptor coactivator of PPARγ, and it is a principal physiological regulator for slow type muscle fiber specification [19–21]. Skeletal muscle–specific PGC-1α knockout mice have significantly impaired glucose tolerance [22], while obese humans have a significantly lower percentage of slow type muscle fibers than humans with lower adiposities [23].

It is strongly suggested that 5-HT may be a key factor with regard to energy metabolism in skeletal muscle, as recent study shows that a 5HTR2 agonist induces the elevation of PGC-1α promoter activity [24]. To verify these hypotheses, we investigated the effect of long-term treatment of mice with peripheral 5-HT on obesity and energy metabolism in skeletal muscle in mice on the high fat diet.

## Materials and Methods

### Animal studies

Male C57BL/6 mice were purchased from Japan SLC (Shizuoka, Japan). All mice were housed in a temperature-controlled facility (23°C) with a 12-hour light/dark cycle and fed a chow diet (14.4 MJ/kg) containing 4.8% fat (Ch) or high-fat diet (17.0 MJ/kg) containing 13.6% fat (F) (CLEA Japan, Inc., Tokyo, Japan). The mice were injected i.p. with serotonin (5-HT) (0.1 mg, 0.5 mg or 1 mg) (Sigma, St. Louis, MO) or phosphate buffered saline (PBS) twice a week between the ages of 5 and 26 weeks. The body weight of mice was measured at the same time as the injections were given. The mice were fasted 12 h before blood and tissues samples were harvested in all experiments. The food intake in each group mice was measured for 5 days when they were 17 weeks of age. The rectal temperature was measured with a thermometer (BAT-7001H; Physitemp Instruments Inc, Clifton, NJ) at 26 weeks of age. The experiments were permitted by the Tohoku University Environmental & Safety Committee and conducted in accordance with the Guidelines for Animal Experimentation of Tohoku University, which have been sanctioned by the relevant committee of the Government of Japan.

### The percentage of body fat and intra-abdominal fat

The proportion of fat in the whole body was determined by Folch’s method. All of the intra-abdominal fat was removed from the body and the weighed. The proportions of total body and intra-abdominal fat were normalized according to body weight.

### Histology of white adipose tissues

Intra-abdominal white adipose tissues were obtained from the mice at 26 weeks of age. These tissues were fixed in 4% paraformaldehyde/PBS, (pH 7.2) and then embedded in paraffin. The staining with hematoxylin-eosin of intra-abdominal white adipose tissues was performed as previously described [13]. The sizes of the intra-abdominal white adipocytes were determined by measuring two hundred cells per sample (n = 5).

### Plasma chemistry analysis

Blood samples were collected from 26 week old mice (n = 7–12) in ice-cold tubes containing heparin (10 unit/tube) (Mochida, Tokyo, Japan), and immediately centrifuged at 20,000 g for 15 min. Plasma samples were stored at -80°C until analysis. All plasma concentrations of hormones and metabolites were measured by commercially available kits supplied by Wako (Osaka, Japan), other than that for leptin and adiponectin, which were supplied by R&D systems (Minneapolis, MN). All procedures were performed according to the manufacturer’s recommendations.

### Glucose and insulin tolerance tests

An i.p. glucose tolerance test was performed in 23 week old mice (n = 6). Glucose (Sigma) was administered i.p. at a dose of 2 mg/g body weight. An i.p. insulin tolerance test was performed in 25 week old mice (n = 6). Insulin (Sigma) was administered i.p. at a dose of 0.225 U/kg body weight. Blood samples were collected from the caudal vein of each mouse at 0, 15, 30, 60, 90 and 120 min after treatment. Plasma glucose and insulin concentrations were measured using the above-mentioned methods.

### Indirect calorimetry

Whole-body energy metabolism was examined using an open-circuit indirect calorimeter (Arco-2000; Arco System, Chiba, Japan). After the system was calibrated against standard gas mixtures, mice were placed in individual acrylic calorimeter chambers with free access to food and water. Energy expenditure, defined as oxygen consumption (VO_2_) and carbon dioxide production (VCO_2_) were measured during a period of 24 h from 16:00 hours at room temperature. The acclimation time was 1 h. Measurements were normalized by body weight.

### Immunohistochemical analysis

Gastrocnemius and soleus muscles obtained from 14 week old mice were frozen in acetone cooled by dry ice. In order to determine skeletal muscle fiber type, the cryosections were cut using a cryostat microtome (Leica, Wetzlar, Germany) and subjected to immunohistochemistry. The sections were immunostained with anti-slow (clone M 8421, 1:600, Sigma) and anti-fast (clone M 4276, 1:300, Sigma) myosin heavy chain monoclonal antibodies, specific markers of type-I and type-II myofibers, respectively. Histofine Simplestain MAX-PO (M) (Nichirei, Tokyo, Japan) was used as the secondary antibody. The proportion of each fiber type was determined in each section of the gastrocnemius and soleus muscles using a photomicroscope photograph (Keyence) and Scion Image software.

### NADH-tetrazolium reductase (NADH-TR) Staining

Fresh-frozen sections of soleus and gastrocnemius muscles in mice at 14 weeks of age of each group were incubated in 0.05 M Tris buffer (pH 7.2) containing NADH (Kohjin Co., Ltd., Tokyo, Japan) and nitoroblue tetorazolium (NBT) (Nacalai Tesque, Inc, Kyoto, Japan) for 30 min at 37°C. Staining was then cleared with 50% acetone and preserved with aqueous mounting medium.

### NAD^+^/NADH ratio assay

Soleus and gastrocnemius muscles were obtained from 14 weeks old mice from each group. About 5 mg of each muscle was used for the analysis of the ratio of NAD^+^/NADH. The concentrations of NAD^+^ and NADH were measured by using a NAD^+^/NADH Quantification Kit (BioVision, San Francisco, CA). The procedure was performed according to the manufacturer’s instructions.

### mRNA expression analysis

Total RNA was extracted from frozen tissue samples using Trizol reagent (Invitrogen, Co., Carlsbad, CA). cDNA was synthesized from total RNA with the Superscript III reverse transcription kit (Invitrogen, Co., Carlsbad, CA) using random primers. The real-time PCR measurement of individual cDNAs was performed using the Thermal Cycler Dice Real Time System Single (Takara Bio Inc., Siga, Japan). After incubation for 10 sec at 95°C, the cDNA was followed by PCR for 40 cycles (95°C, 5 sec: 60°C, 30 sec). SYBR green fluorescence was detected at the end of each cycle to monitor the amount of PCR product formed during that cycle. At the end of each run, the melting curve profiles were recorded. The standard curve of each product followed the calculation of the respective gene expressions. Values were normalized to those of 18S ribosomal RNA. The primer sequences are listed in Table 1.

**Table 1:** Primers used in quantitative real-time PCR analysis for determining expression of mRNA.

Genes		Primer sequence	Product size (bp)
Total PGC-1α	F	CCGTAAATCTGCGGGATGATG	114
	R	CAGTTTCGTTCGACCTGCGTAA	
PGC-1α-a	F	GCTTGACTGGCGTCATTCG	59
	R	ACAGAGTCTTGGCTGCACATGT	
PGC-1α-b	F	GACATGGATGTTGGGATTGTCA	61
	R	ACCAACCAGAGCAGCACATTT	
PGC-1α-c	F	AGTGACATGGATGTTGGGATTG	66
	R	GAATGCCTCCGGTTACTCACTT	
D2	F	CTGCTCAGTCTGTGGTTGGATGTAG	91
	R	TGCACCATGACCCAAATGTTC	
UCP-3	F	CTGAAGATGGTGGCTCAGGA	144
	R	CCGCAGTACCTGGACTTTCATTA	
NRF1	F	GCTTGTGAAGTCCAGGGACAGAG	110
	R	AGGGCGTTTACCTGCCTGTG	
PPARγ	F	TGTCGGTTTCAGAAGTGCCTTG	122
	R	TTCAGCTGGTCGATATCACTGGAG	
Cyt-C	F	CCATTACCCTGGTGTGGCTTTC	70
	R	ACAGCAGCCATTAGCTACTTCATCA	
COX4	F	TATGCTTTCCCCACTTACGC	159
	R	CTGGATGCGGTACAACTGAA	
HSL	F	TCCTGGAACTAAGTGGACGCAAG	93
	R	CAGACACACTCCTGCGCATAGAC	
CPT-1b	F	GAGACAGGACACTGTGTGGGTGA	107
	R	TGGTACGAGTTCTCGATGGCTTC	
5HTR1A	F	CCTGCCACATGAAGCCATTG	136
	R	GGTGTGGACACCCTACAGGCTTA	
5HTR1B	F	TGGCCGCATCTATGTGGAAG	88
	R	TATCAACTGGGCTCGGGTCAA	
5HTR1D	F	CATCTGCAGGGACTCTTGTTGG	124
	R	CGCTTGTCGAAAGTCTTCGTTG	
5HTR1F	F	CAGATCGGAACTGAAGCATGAGAA	97
	R	ACCCAAGATCAATCCCAGGGTAG	
5HTR2A	F	TAGCCGCTTCAACTCCAGAACC	117
	R	AAGACCTTCGAATCATCCTGTAGCC	
5HTR2B	F	CGGGCTACTGCATTCATCAAGA	122
	R	AGCTCACAGGTGACATTGTGTGG	
5HTR2C	F	CATGTTCCCAGTAACTGTGTTTCCA	120
	R	GCTCACTCCAAGGTGTGCAAGTAG	
5HTR3A	F	AGCCAACAAGACTGATGACTGCTC	74
	R	CAACATGGCTGCAGTGGTTTC	
5HTR3B	F	TTCAGGGTCAACATGTCTGATGAAG	85
	R	GGGCCATGCAGACGGTAAAG	
5HTR4	F	AAGTACATGTGTGCCTGCTGTTGAG	97
	R	TAGCCAACCAGTTCATGACACCA	
5HTR5A	F	TTTACAGGGCGGCGAAAT	118
	R	CGGACCGTGAACACCATCT	
5HTR5B	F	AGTTTCGATTCGGTCGCAGA	82
	R	TCAGACTCCGGAGGTGCTTC	
5HTR6	F	CTGACCACCAAGCATAGCAGGA	162
	R	CAGCCATGTGAGGACATCGAA	
5HTR7	F	CTAACGCACAATTCCCATGCTTC	142
	R	GCAACACATTCAACACGATGCTTAC	
18S	F	CGGCTACCACATCCAAGGAA	125
	R	GCTGGAATTACCGCGGCT	

F: Forward primer, R: Reverse primer

### Effect of serotonin receptor antagonists

Ketanserin (Sigma), an antagonist for 5HTR2A, was dissolved in 0.1 M HCl, diluted with PBS, and administered in a dosing volume of 0.1 mg/mouse. SB-204741 (Tocris Bioscience, Bristol, UK), an antagonist for 5HTR2B, was dissolved in DMSO, diluted with PBS such that the final concentration of DMSO was 0.1%, and administered in a dosing volume of 0.08 mg/mouse. SB-269970 (Sigma), 5HTR7 antagonist, and methysergide (Sigma), a 5HTR1, 2 and 7 antagonist, were dissolved in PBS and administered in a dosing volume of 0.6 and 0.1 mg/mouse, respectively. All antagonists were i.p. injected at 30 min before the injection of 1 mg 5-HT. After 120 min, samples were collected from skeletal muscle.

### Statistical analysis

Values are reported as means ± SE. Statistical analyses were performed using Student's t test or one-way and two-way ANOVA followed by Tukey’s test to evaluate statistical differences among the groups. P values less than 0.05 were considered statistically significant.

## Results

### 5-HT inhibits weight gain and adiposity of mice fed a high fat diet

**Fig 1:**
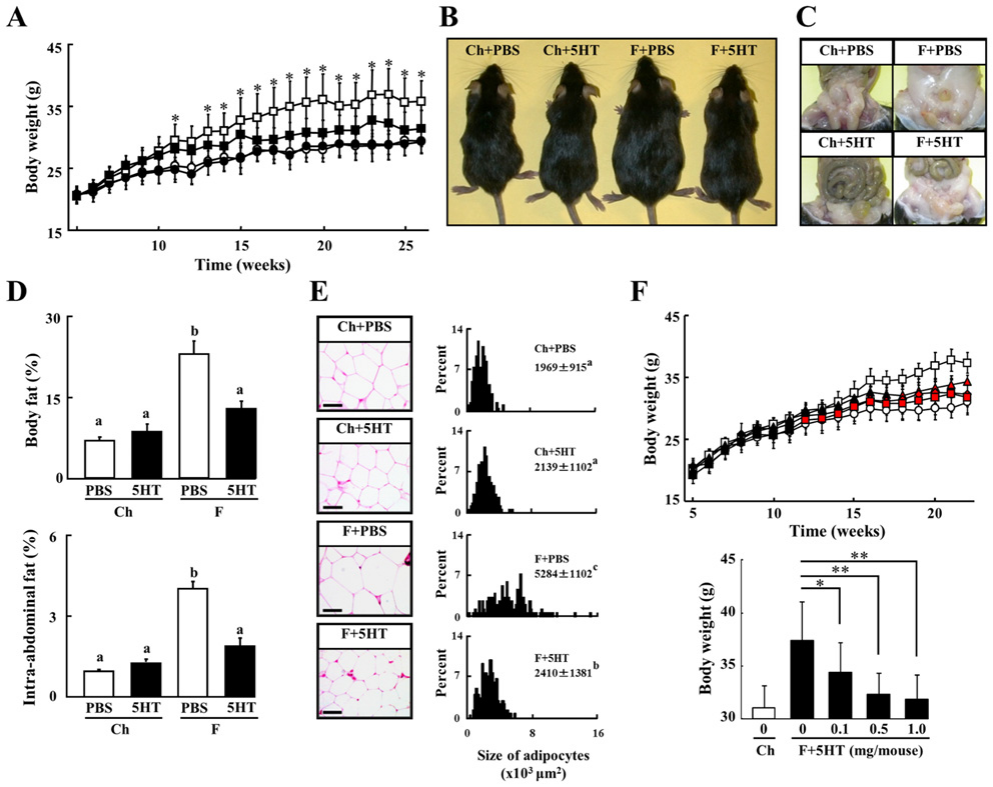
Effect of 5-HT on Adiposity of Mice Fed a High Fat Diet. A, Body weight of male C57BL/6 mice fed a chow diet (Ch) or a high fat diet (F) were measured over 26 weeks (n = 12 to 20). Mice were intraperitoneally injected with PBS or 5-HT (1 mg/mouse) twice a week. Open circles, chow plus PBS (Ch+PBS); filled circles, chow plus 5-HT (Ch+5HT); open squares, F diet plus PBS (F+PBS); filled squares, F diet plus 5-HT (F+5HT). B and C, Photographs are the gross appearance (B) and the abdominal cavity (C) of representative mice from each group at 26 weeks of age. D, The proportion of whole body fat (n = 5) and intraabdominal fat (n = 10) of each group of mice at 26 weeks of age. E, The adipose tissues of mice at 26 weeks of age were stained with hematoxylin and eosin. Bars in histological sections indicate 50 μm. Figs show the size distribution of adipocytes. Data are means ± s.d. Figs with a different superscript letter are significantly different (p<0.05). F, The change in body weight of C57BL/6 mice treated with several doses of 5-HT were measured over 22 weeks (n = 14 to 20). The body weight of mice at 22 weeks of age shows the concentration dependent effect of 5HT. Open circles, Ch+PBS; open squares, F+PBS; filled symbols, F+5HT at a concentration of 0.1 mg (▲), 0.5 mg (◆) and 1.0 mg/mouse (■). Red colored symbols show significance in F+5HT mice against F+PBS mice (p<0.05). Data are means ± s.e. All data were analyzed by two-way ANOVA without (F). Data in (F) was analyzed by one-way ANOVA. Columns with a different letter are significantly different (p<0.05). *p<0.05, **p<0.01 indicates significance in F+5HT mice against F+PBS mice.

Mice were fed a chow (Ch) diet or a high fat (F) diet and injected i.p. with 1 mg of 5-HT twice a week between the ages of 5 and 26 weeks. Mice on the F diet gained significantly more weight than the Ch fed mice at 8 weeks of age, and became markedly obese by 26 weeks of age. 5-HT treated mice on the F diet gained weight until 10 weeks of age, however, their weight gain tailed off after 13 weeks of age and they approached the average weight of the Ch fed mice (Fig 1A). Interestingly, the administration of 5-HT did not affect the weight gain of the Ch fed mice. Although the high fat diet increased the whole body mass (Fig 1B), the accumulation of intra-abdominal fat (Fig 1C), and the percentages of total body fat and intra-abdominal fat (Fig 1D), those in 5-HT treated mice on the F diet were not different from the control mice on the Ch diet. The administration of 5-HT completely prevented the enlargement of the intra-abdominal adipocytes in mice on the F diet (Fig 1E). The anti-obesity activity of 5-HT in mice on the F diet was observed from 18, 16 and 12 weeks of age following 5-HT injection of 0.1, 0.5 and 1 mg, respectively (Fig 1F). The effect was concentration-dependent when analyzed at 26 weeks of age.

**Fig 2:**
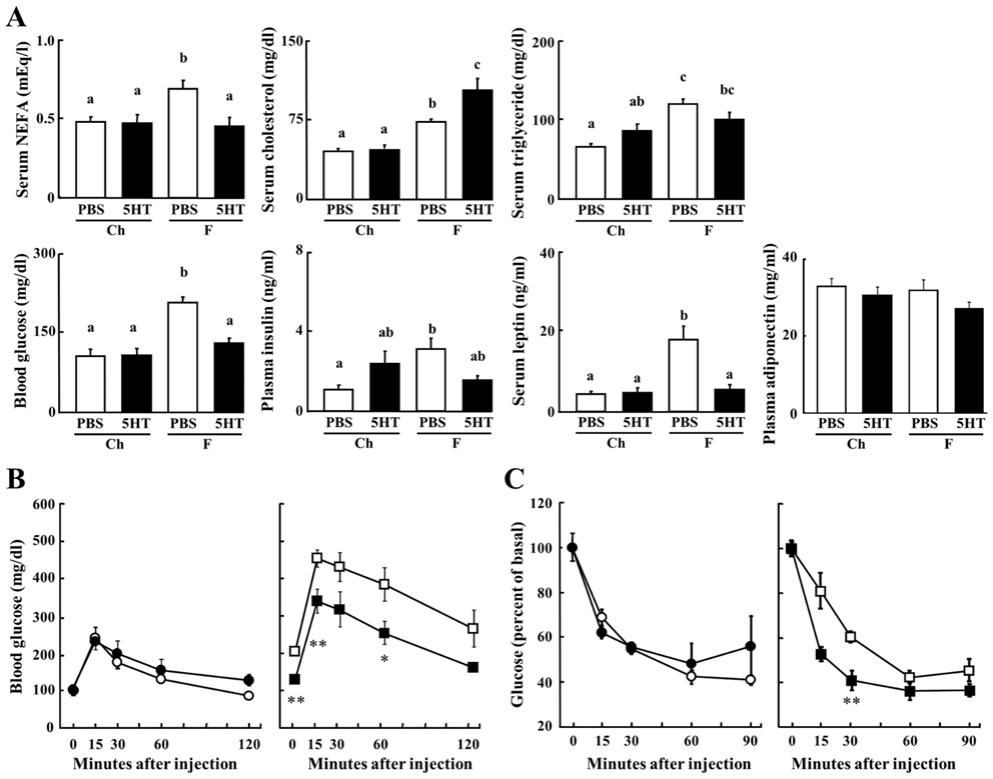
Metabolic Effect of 5HT on a High Fat Diet. A, The concentrations of blood NEFA, cholesterol, triglyceride, glucose, insulin, leptin and adiponectin were measured in mice aged between 21 and 26 weeks of age (n = 7–12). B and C, Glucose tolerance (B) and insulin tolerance (C) tests were performed in each group of mice at 21 weeks of age (n = 7). Circles, Ch+PBS; filled circles, Ch+5HT; squares, F+PBS; filled squares, F+5HT. Data are means ± s.e. Data in (A) were analyzed by two-way ANOVA. Data in (B) and (C) were analyzed by Student's t test. Columns with a different letter are significantly different (p<0.05). *p<0.05, **p<0.01 indicates significance in F+5HT mice against F+PBS mice.

The blood concentrations of NEFA, cholesterol, triglyceride, glucose, insulin and leptin increased in F diet-fed control mice following their body weight gain (Fig 2A). The concentrations of these hormones and metabolites, other than for cholesterol, were maintained in 5-HT treated mice on the F diet at the levels seen in the mice on the Ch diet. Although 5-HT has been reported to inhibit adiponectin expression in the 3T3L1 adipocyte [25], there were no differences in blood adiponectin levels between each group. Intraperitoneal glucose and insulin tolerance tests show that 5-HT treatment protected against the hyperglycemia and an elevation in insulin resistance observed in mice on the F diet (Fig 2B and 2C).

**Fig 3:**
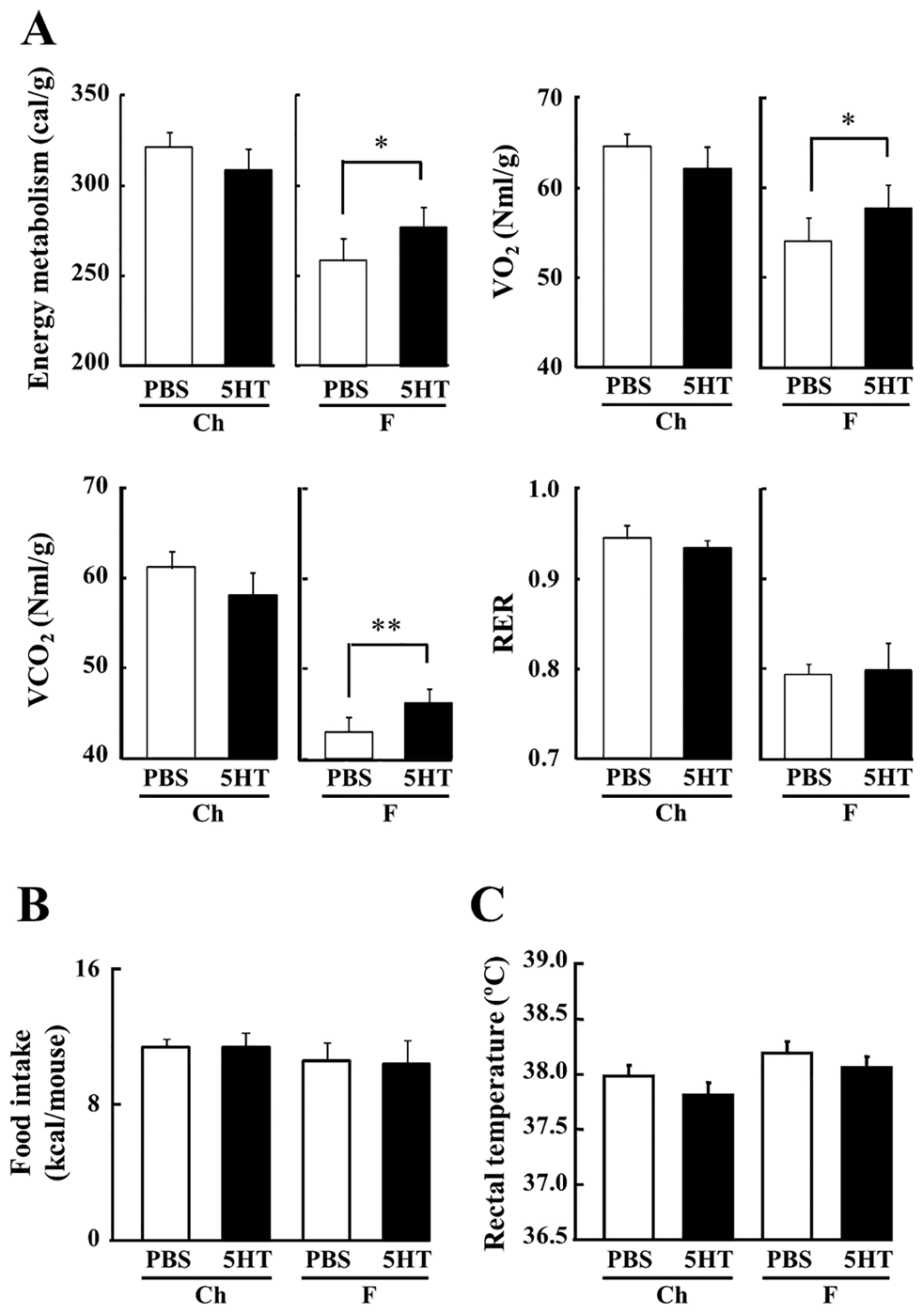
Effect of 5-HT on Energy Metabolism. A, Energy expenditure, oxygen consumption, production of carbon dioxide and respiratory exchange ratio were measured for 24 h in each group mice at 21 to 23 weeks of age (n = 5–6). The acclimation time was 1 h. These values were normalized to body weight. B, The food intake of each group of mice was measured for 1 day at 17 weeks of age (n = 6). C, The rectal temperatures of each group mice at 22 weeks of age are indicated (n = 14). Data are the mean ± s.e. Data were analyzed by Student's t test (A) and two-way ANOVA (B). Columns with a different letter are significantly different (p<0.05). *p<0.05, **p<0.01 indicates significance in 5-HT-treated mice against PBS-treated mice.

Using indirect calorimetry, energy metabolism, O_2_ consumption, CO_2_ production and respiratory exchange ratio (RER) were higher in 5-HT injected mice on the F diet, but 5-HT did not affect these measures in mice on the Ch diet (Fig 3A). In contrast, 5-HT did not affect food intake and rectal temperature in mice on either diet (Fig 3B and 3C). These results demonstrate that the increase in energy expenditure following 5-HT treatment ameliorates the obesity and diabetes of mice on the F diet, without any change of food intake.

### 5-HT elevates the proportion of slow type muscle fibers in the skeletal muscle of mice on the high fat diet

**Fig 4:**
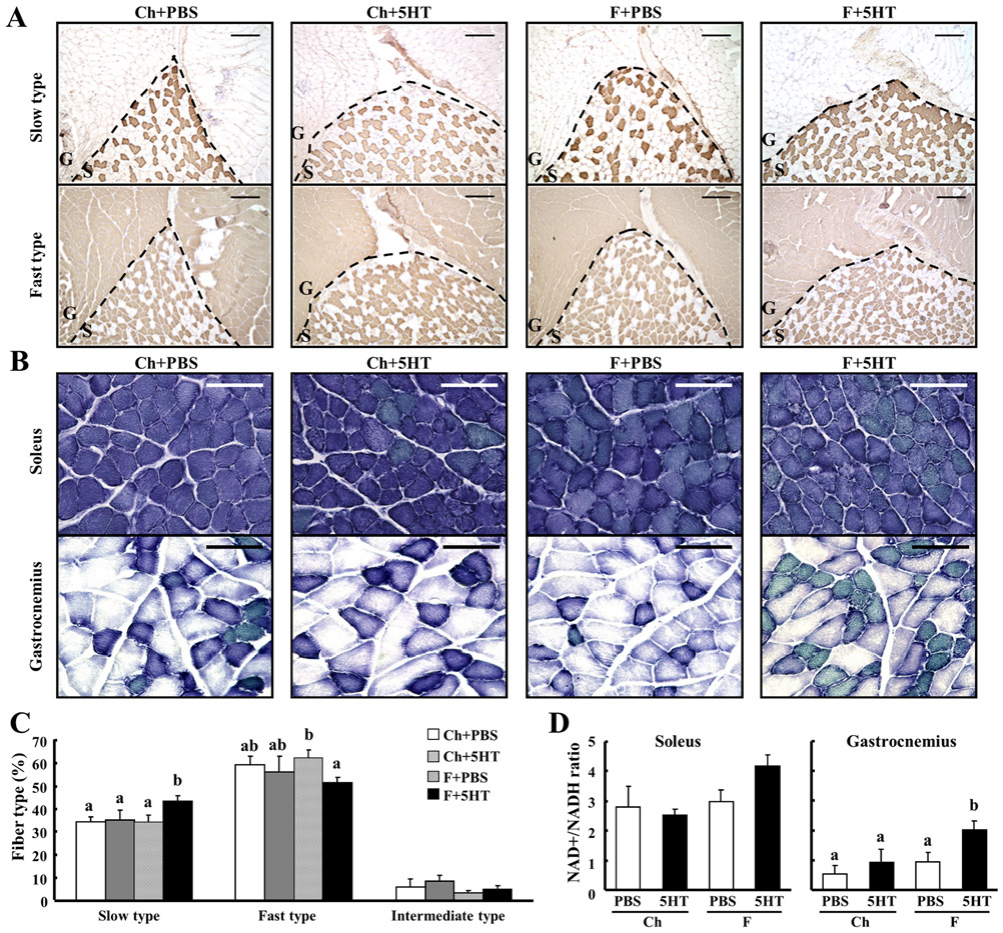
5-HT elevates the proportion of slow type muscle fibers in the skeletal muscle of mice on the high fat diet. A, Mice fed a chow diet (Ch) or a high fat diet (F) were i.p. injected with PBS or 5-HT (1 mg/mouse) twice a week. After 8 weeks of treatment, the frozen sections of soleus and gastrocnemius muscles of 14 weeks of age mice were immunostained using anti-slow and anti-fast myosin heavy chain antibodies, specific markers of myofiber type-I and type-II, respectively (n = 5). S and G in (A) indicate the positions of soleus and gastrocnemius muscles, respectively. Bars in histological sections indicate 200 μm. B, The activities of oxidative enzyme in soleus and gastrocnemius muscles of mice at 14 weeks of age of each group were examined by enzymatic staining using NADH-tetrazolium reductase (NADH-TR) shown with a blue precipitate. Bars in histological sections indicate 50 μm. C, The proportions of slow, fast and intermediate type muscle fibers in soleus muscle of each group of mice were calculated by evaluating the type of all soleus muscle fiber in each immunostaining section in (A) (n = 5). D, Intracellular levels of NAD-to-NADH ratio were measured in soleus and gastrocnemius muscles of mice at 14 weeks of age (n = 5–7). Data are the mean ± s.e. Data were analyzed by two-way ANOVA. Columns with a different letter are significantly different (p<0.05).

In order to identify the target tissues of the effect of 5-HT on energy metabolism, we focused on skeletal muscle, and especially the slow muscle fiber type, which has been shown to principally metabolize fatty acids by oxidative means. In the soleus muscle of mice on the F diet at 14 weeks of age, 5-HT significantly increased the proportion of slow muscle fibers and decreased the proportion of fast muscle fibers (Fig 4A and 4C). However, these effects were not seen in mice on the Ch diet. In addition, increases in the proportion of oxidative muscle fibers (darker blue) and the NAD+/NADH ratio were observed in gastrocnemius muscle of 5-HT treated mice on the F diet, though there was no difference between treatments in the soleus muscle (Fig 4B and 4D).

**Fig 5:**
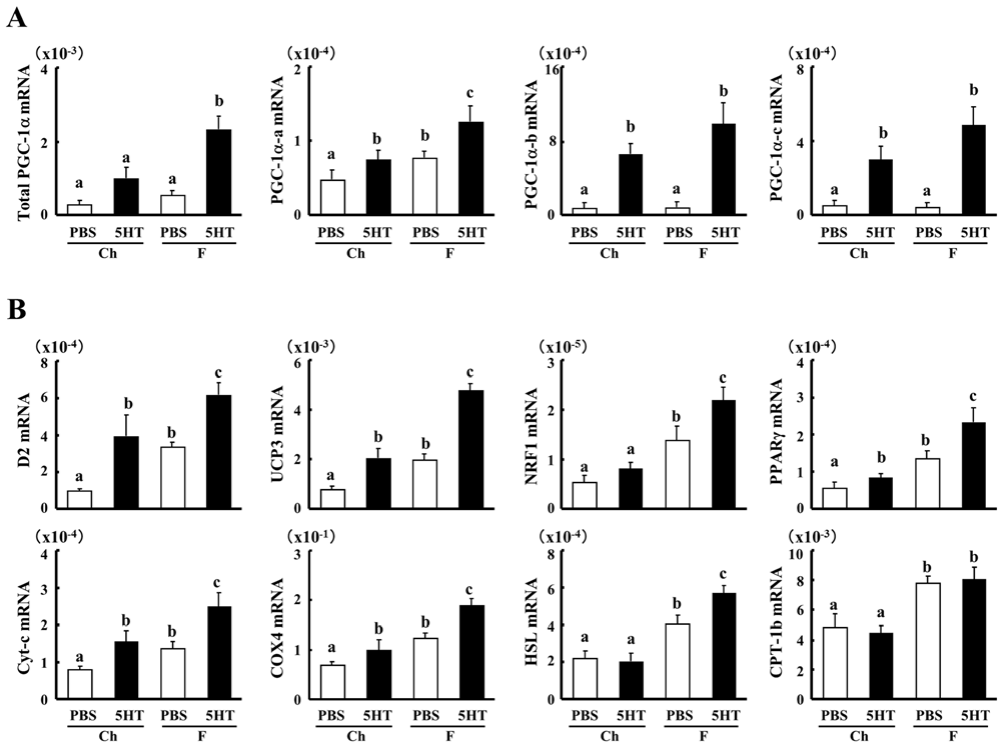
Effect of 5-HT on mRNA expression in soleus muscle. The relative mRNA expression levels of Total PGC-1α, PGC-1α-a, PGC-1α-b, PGC-1α-c, D2, UCP3, PPARγ, NRF1, Cyt-c, COXIV, HSL and CPT-1b were analyzed in soleus muscle of each group of mice at 14 weeks of age by quantitative real-time RT-PCR (n = 6–8). Data are the mean ± s.e. Data were analyzed by two-way ANOVA. Columns with a different letter are significantly different (p<0.05).

PGC-1α is a master regulator that promotes mitochondrial biogenesis and a fiber switch to slow muscle fiber type in skeletal muscle [19–21]. It has been recently reported that PGC-1α mRNA has three isoforms, PGC-1α-a, b and c [26]. Therefore, we measured the mRNA expressions of PGC-1α isoforms (Fig 5A). 5-HT increased the expression of total PGC-1α in soleus muscle of mice on the F diet, following the dramatic elevation of PGC-1α-b and c expressions.

Additionally, the expressions of key genes in energy homeostasis, such as type 2 iodothyronine deiodinase (D2) and uncoupling protein (UCP) 3 were elevated by 5-HT injection in both diet groups (Fig 5B). 5-HT selectively increased the mRNA expressions of peroxisome-proliferator-activated receptor γ (PPARγ), hormone-sensitive lipase (HSL) and the genes related to mitochondrial biogenesis and activities, such as nuclear respiratory factor (NRF) 1, cytochrome c (Cyt-c), and cytochrome c oxidase subunit (COX) IV in mice on the F diet, other than carnitine palmitoyltransferase 1b (CPT-1b). These data suggest that 5-HT may be able to increase the oxidative muscle fiber type and the energy metabolism of skeletal muscle.

### Mechanism of elevation of energy expenditure in skeletal muscle by 5-HT

**Fig 6:**
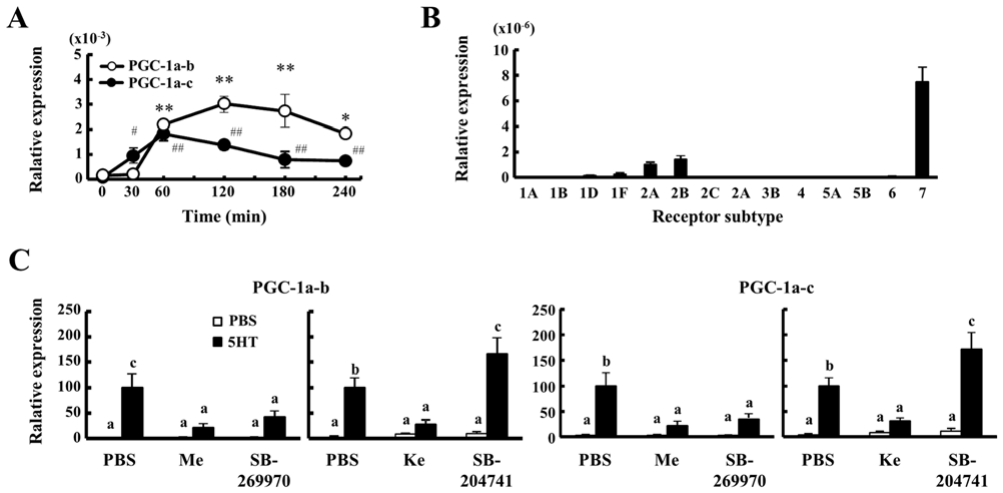
Elevation of mRNA Expressions of PGC-1α-b and PGC-1α-c by 5HT. A, In 8 weeks of age mice, the mRNA expression levels of PGC-1α-b and PGC-1α-c were measured in soleus muscle and after i.p. injection of 1 mg 5-HT by real time PCR (n = 6–8). B, The relative mRNA expression levels of 5-HTR were measured in soleus muscle of mice at 8 weeks of age by using real time PCR (n = 6–8). C, At 30 min before the administration of 1 mg 5-HT, mice were injected with several 5-HTR antagonists: Methysergide (antagonist for 5-HTR1, 2 and 7, 0.1 mg/mouse), SB-269970 (5-HTR7, 0.6 mg), Ketanserin (5-HTR2A, 0.1 mg/mouse) and SB-204741 (5-HTR2B, 0.08 mg/mouse). The mRNA expressions of PGC-1α-b and PGC-1α-c in soleus muscle were determined at 120 min after 5-HT injection by real time PCR (n = 6–8). The mRNA expression levels in PBS-pretreated mice injected with 5-HT are designed as 100. Values in soleus muscle were normalized to those for 18S. Data are means ± s.e. Data were analyzed by Student's t test (A) and one-way ANOVA (C).*, P<0.05; **, P<0.01 relative to basal values at 0 min of PGC-1α-b. #, P<0.05; ##, P<0.01 relative to basal values at 0 min of PGC-1α-c. Columns with a different letter are significantly different (p<0.05).

In order to clarify the mechanism by which PGC-1α-b and c are elevated by 5-HT, we measured the mRNA expression levels in soleus muscle of mice at 8 weeks of age after a single injection of 5-HT. The expressions of these mRNA were dramatically elevated in soleus muscle after the injection of 5-HT (Fig 6A). In addition, soleus muscle had higher expressions of 5HTR2A, 2B and 7 (Fig 6B). In order to determine what kinds of 5HTR were related to the elevation of PGC-1α-b and c gene expressions in soleus muscle, mice were pre-treated with several 5-HTR antagonists at 30 min before 5-HT injection. In soleus muscle, the elevation of these mRNA expressions at 120 min after the 5-HT injection were significantly inhibited by methysergide (5HTR1, 2 and 7 antagonist), ketanserin (5HTR2A antagonist) and SB-269970 (5HTR7 antagonist), but not SB-204741 (5HTR2B antagonist) (Fig 6C). These data indicate that 5-HT increases the mRNA expressions of PGC-1α-b and c through 5-HTR2A and 7 in soleus muscle.

## Discussion

Neural 5-HT is believed to modulate numerous sensory, motor and behavioral processes, and be also involved in the control of feeding in the mammalian nervous system. It has been previously reported that neural 5-HT has a suppressive effect on food intake and tends to decrease body weight gain [5–10]. 5HTR2C, a prominent G-protein-coupled receptor, is widely expressed throughout the brain and spinal cord and has been proposed to mediate numerous central nervous system actions of 5-HT [27]. 5HTR2C deficient mice are overweight as a result of an abnormal control of feeding behavior, establishing a role for this receptor in the serotonergic control of appetite [8, 28]. In contrast, peripheral 5-HT did not cause any change in food intake behavior in mice in the present experiment, not only on the F diet but also on the Ch diet. In addition, the peripheral injection of 5-HT also did not cause any change in the rate of body weight gain in mice on the Ch diet. A study on 5-HT signaling in *Caenorhabditis elegans* reported that serotonergic regulation of fat was molecularly distinct from feeding regulation, and that obesity and thinness were not solely determined by feeding behavior [14]. As peripheral 5-HT is thought to be unable to pass the blood-brain barrier, 5-HT likely had an independent mechanism affecting obesity in the periphery, and not through any central nervous system involvement [3, 4].

Mice on the F diet gained weight until 10 weeks of age with or without the peripheral 5-HT treatment, though 5-HT inhibited their body weight gain after 13 weeks of age. However, it remains unclear why 5-HT decreased body weight gain in mice on the F diet, but not on the Ch diet. In skeletal muscle, 5-HT intriguingly induced an elevation of energy expenditure-related gene expressions, as well as morphological changes in the mice, on the F diet more so than on the Ch diet. It has been reported that the alterations in fat accumulation in the intra-abdominal organs, such as visceral adipose tissue and the liver, induce hormonal and neuronal signaling pathways, resulting in cooperative metabolic regulation among tissues/organs throughout the body [29]. In addition, hepatic activation of extracellular regulated kinase (ERK) signaling induced pancreatic β cell proliferation through a metabolic relay from the liver to the pancreas [30]. 5-HT also stimulates the contraction of the gallbladder and the excretion of bile, and accelerates the metabolism of lipid and the function of liver by increasing the concentration of bile acids in circulation [13]. These reports indicate that metabolic regulation in mammals requires communication between multiple organs and tissues, and that 5-HT may prevent or even reverse fat accumulation and body weight gain after the consumption of surplus energy following F diet feeding.

In this study, we confirmed that treatment with 5-HT induced a shift of the profile of muscle fiber type from fast/glycolytic type to slow/oxidative type in soleus muscle, especially in mice on the F diet. In addition, the injection of 5-HT increased the mRNA expressions of PGC-1α-b and c and genes related to the oxidation of fatty acids in skeletal muscle in mice on the Ch diet, and dramatically so for mice on the F diet. Recent studies demonstrated that transgenic mice overexpressing PGC-1α-b or c in skeletal muscle induced higher gene expressions involved in mitochondrial biogenesis and fatty acid oxidation [26]. Exercise capacity and oxygen uptake were also increased in skeletal muscle in mice over-expressing PGC-1α-b [31]. The expressions of PGC-1α-b and c mRNA in skeletal muscle were remarkably elevated by exercise [26]. Additionally, 5-HT controls the biogenesis of mitochondria by increasing the PGC-1α promoter activity through 5-HT receptors in renal proximal tubular cells [24]. Taken together, our data indicate that 5-HT may prevent fat accumulation and body weight gain by morphological changes of skeletal muscle through an elevation of PGC-1α-b and c expressions in mice with surplus energy during F diet feeding. However, there are few studies on an effect of an intestinal hormone on the ratio of muscle fiber type without the effect of exercise or the knockout of one or more genes. Therefore, it is a novel finding that 5-HT can induce a shift from glycolytic to oxidative metabolism in muscles.

Different findings have been found with regard to 5-HT function in glucose and lipid metabolism between genetic and treatment studies with 5-HT. A recent study demonstrated that gut-derived 5-HT signaling through the 5HTR2B promotes lipolysis in adipocytes and gluconeogenesis in hepatocytes, but inhibits glucose uptake in hepatocytes in the TPH1 deficiency mice model [16]. Genetic inhibition of Tph1 protects or reverses the development of F diet induced obesity and dysglycemia via activation of UCP1-mediated thermogenesis in brown adipose tissue [17]. Additionally, 5-HT suppresses glycogen synthesis at micromolar levels but promotes it at nanomolar levels in hepatocytes by serotonergic mechanisms [32]. In contrast, 5-HT increases net hepatic glucose uptake in hyperglycemic and hyperinsulinemic conditions [33]. Our previous study shows that 5-HT injection induces an increase in the hepatic glycogen content, and the decrease of hepatic triglyceride content [13]. In this study, in mice on the F diet at 14 weeks of age, 5-HT significantly decreased the liver weight and hepatic triglyceride content, however, there was no change in the expressions of β oxidation related gene in the liver of mice injected with 5-HT (data not shown). These data suggest that 5-HT indirectly decreases the fat accumulation in the liver after an elevation of energy expenditure by 5-HT. Fourteen 5HTRs are identified and 5-HT is thought to cause its effects through these membrane bound receptors [34]. In this report, we have observed that an injection of 5-HT induced an elevation of PGC-1α-b and c mRNA expressions in soleus muscle through 5HTR2A and 7. Taken together, these results suggest that 5-HT may have an important role in glucose and lipid metabolism and energy expenditure in some tissues through the 5HTRS.

The studies on the molecular mechanism of up-regulation of PGC-1α show that the cAMP response element (CRE) sequence was required for contractile-induced activation of PGC-1α promoter [35], and that the transducers of CRE-binding protein (CREB) and coactivators of CREB (TORCs) markedly activated PGC-1α transcription and mitochondrial biogenesis [36]. Because TORC is activated by cAMP [37], cAMP is mainly involved in an increase of PGC-1α expression. As 5-HT stimulates cAMP accumulation in 5HTR7-transfected CHO cells [38], 5-HT may be able to increase the expression of PGC-1α directly through 5HTR7. However, almost all agonists and antagonists of 5HTR can pass the blood-brain barrier; 5-HT studies in the central nervous system have received a lot of attention in recent years. Therefore, it is difficult for us to pinpoint the peripheral effect of 5-HT completely. There is a clear need for the development of 5HTR agonists and antagonists that act only in the periphery in order for further progress in the understanding of the other peripheral functions of 5-HT.

In conclusion, we have elucidated that the intraperitoneal injection of 5-HT prevents obesity by inducing an increase in the activity of mitochondria and an elevation of energy metabolism in skeletal muscle of mice on a high fat diet. We speculate that peripheral 5-HT action may well offer new strategies for developing therapeutic drugs for the treatment of metabolic diseases such as hyperlipidemia, diabetes and obesity.
